# State and Non-State Conflict-Related Sexual Violence Against Women and Girls During the Northern Irish ‘Troubles’: A Systematic Review

**DOI:** 10.1177/15248380251366267

**Published:** 2025-11-08

**Authors:** Orla Kerrigan, Conor Murray, Jennifer Ferguson

**Affiliations:** 1 Ulster University, Belfast, UK; 2 Teesside University, Middlesbrough, Tees Valley, UK

**Keywords:** gender-based violence, sexual assault, victims/survivors < sexual assault, sexual harassment, trauma

## Abstract

In the international context, there is a prevailing perception that conflict-related sexual violence (CRSV) did not occur during the ethno–nationalist conflict in Northern Ireland (NI), commonly referred to as the ‘Troubles.’ This systematic review shows that CRSV did occur and was widespread and systemic. The review focused on women and girls of all ages in NI during the Troubles. It excluded CRSV outside NI. We searched PyschInfo, EMBASE, and APA Physcarticles (via OVID); PUBMED, CINAHL Ultimate, Criminal justice abstracts, Medline, peace research abstracts, and women’s study international (via EBSCOHost); the ProQuest social sciences premium collection (25 databases including the National Criminal Justice Reference Service Abstracts); the Cochrane Library; Scopus; Web of Science; the first 10 pages of Google Scholar; and specialist websites (notably of Amnesty International, the UK Ministry of Justice, the NI Department of Justice, and the Office of the United Nations High Commissioner for Human Rights [OHCHR]). Of 4,061 database results, 300 Google Scholar results, and 5,187 ‘reference list checking’ searches, 47 publications met the inclusion criteria. Fourteen publications were identified through database searches, 8 publications through Google Scholar, and 25 publications through reference searches. The publications illuminate the experiences of women and girls in NI who suffered conflict-related sexual, physical, verbal, and psychological harassment, perpetrated by state or non-state actors, in community or criminal justice settings. Available accounts and testimonies show that violence was pervasive, widespread, and created intense fear and vulnerability.

## Introduction

Since its creation under the Government of Ireland Act (1920), Northern Ireland (NI) has experienced persistent tension and political violence. These escalated significantly between 1968 and 1998, a period colloquially known as the ‘Troubles.’ The Troubles was a conflict between the Catholic/nationalist/republican (CNR) community and the Protestant/unionist/loyalist (PUL) community, and paramilitary organisations associated with these communities. However, it also involved state security forces, particularly the British Army and NI’s police force, the Royal Ulster Constabulary (RUC) (Melaugh, 2009). During the Troubles, the RUC and British Army were closely intertwined, co-operating on policing, intelligence gathering, and joint security operations. There was significant overlap between the British Army and the Ulster Defence Regiment (UDR), a British Army unit recruited in NI. Many RUC officers also served as members of the UDR (Sanders, 2011).

This dynamic shaped both the strategies of conflict and the lived experiences of those affected by it. The Good Friday Agreement (GFA) (1998) initiated a new era in NI. It promised peace after an ethno–nationalist conflict that had lasted more than 30 years and that in NI alone was the direct cause of approximately 3,532 deaths (Sutton, 2022), more than 40,000 injuries (Manktelow, 2007), and half a million acknowledged ‘victims’ (defined by the government as individuals who were directly affected by ‘bereavement,’ ‘physical injury’ or ‘trauma’ as a result of the conflict) (O’Leary & McGarry, 1993). For these victims, the GFA promised redress and to “acknowledge and address the suffering of victims of violence” (Department of Foreign Affairs, 1998: 18). However, despite clear feminist scholarship (Aretxaga, 2000, 2001; Corcoran, 2006) and activism by Irish republicans (Women in struggle, 1992a, 1992b) to illuminate the prevalence of CRSV in NI, the peace process remained state-centric and institution-led, with the GFA focusing solely on ‘conventional forms of military violence.’ It did not acknowledge or address CRSV or gender-based violence (GBV) more generally (O’Rourke, 2013; O’Rourke & Swaine, 2017).

The United Nations defined CRSV as “incidents or patterns of sexual violence against women, men, girls or boys occurring in a conflict or post-conflict setting that have direct or indirect links with the conflict itself or that occur in other situations of concern such as in the context of political repression” (United Nations, 2011: 3). While this definition captures direct forms of sexual violence such as rape and sexual assault, it fails to encompass the structural and systemic gendered abuses that women and girls experienced during the Troubles, including paramilitary surveillance, coercive control, and policing of their sexuality (Mukungu, 2022). For example, strip searching of women in prison was not only a humiliating practice but also a tool of political oppression (Aretxaga, 1997; Wahidin, 2016). Outside prison, paramilitary groups enforced behavioural norms and curtailed women’s autonomy, reinforcing patriarchal control within communities (Fairweather et al., 1984; McCafferty, 1981; O’Keefe, 2013). This systematic review adopts Swaine’s (2022) broader conceptualisation of CRSV, which recognises that CRSV extends beyond direct physical acts to include mechanisms of intimidation, coercion, and gendered oppression.

CRSV has been described in several conflicts, including Rwanda (Fabri et al., cited in Burnet, 2015; Nowrojee, 1996), the Democratic Republic of Congo (Marks, 2013; Pereznieto et al., 2014), Sierra Leone (Meger, 2010), and Bosnia (Snyder et al., 2006). Research initially attributed CRSV to unfulfilled and uncontrollable sexual urges of soldiers, as well as to a breakdown of ordinary social rules and norms (Meger, 2010). St. Germain (2012) subsequently pointed out that CRSV was also used as a biological weapon to achieve political power, cause displacement, and humiliate enemies. Since then, most case studies and most CRSV research have focused on ‘strategic rape,’ understood to be the deliberate and systematic rape of civilians during armed attacks, used both as a weapon and a ‘spoil’ of war. CRSV occurs in a variety of conflicts for many different reasons. Seifert (1994) identified five understandings of sexual violence in conflict. (a) Sexual violence is simply an integral component of warfare. (b) It is an element of male-to-male communication, which usually involves the symbolic humiliation of opponents by attacking their masculinity. (c) Attackers commit sexual violence to reaffirm their masculinity. (d) Attackers use it to destroy the culture of opponents by tainting their bloodlines. (e) It is an expression of misogyny.

Goldstein (2001) and Stern and Nystrand (2006) discussed the symbolism of CRSV at the macro level. They argued that CRSV may be used to terrorise enemy women and humiliate and ‘emasculate’ enemy men by ‘breaking’ or ‘destroying’ the women and proving their men are incapable of protecting them. It has also been suggested that CRSV symbolically represents a conquering of the ‘motherland.’ Yuval-Davis and Anthias (1989) emphasised that, ideologically, nationalisms restrict the roles of women to childbearing and the reproduction of culture. In these terms, armed groups use CRSV to conquer enemy women and simultaneously exterminate the purity of their society’s culture and bloodline, now ‘tainted’ with enemy blood. In this manner, women’s bodies may become symbolic battlegrounds (McClintock, 1995).

CRSV, like all forms of sexual violence, is a profoundly intimate violation of a victim’s human rights and bodily autonomy. The assault can cause physical trauma and result in long-term complications, such as chronic pelvic pain, pelvic organ dysfunction, infertility, incontinence, and even death. It can also lead to unwanted pregnancies and associated risks, as well as the transmission of sexually transmitted infections, either accidentally or purposefully, which may have lasting health consequences, including infertility and death (Kinyanda et al., 2010). CRSV often has profound physical, psychological, and social impacts that are distinct from those caused by other forms of sexual violence. Victims frequently suffer from anxiety, depression, and post-traumatic stress disorder. These physical and psychological effects are often compounded by social effects, including a sense of shame and isolation, which are particularly prevalent in morally conservative communities (Rubini et al., 2023). In such cases, the perpetrator often represents an ongoing source of shame and embarrassment, intensifying the victim’s risk of community rejection and isolation. In some cases, victims may be at risk of honour-based violence, punishment, or even execution at the hands of their own relatives for the perceived disgrace inflicted upon their families (Bastick et al., 2007). Similarly, it is more likely that children conceived as a result of CRSV will be unwanted and may become victims of infanticide or neglect, or face exclusion from their communities (Mukwege & Nangini, 2009).

Despite the GFA’s promise of peace, the legacy of the conflict persists in many unaddressed ways. CRSV in NI has inflicted unique harms that remain largely unacknowledged and unresolved. While documented cases of domestic abuse (McWilliams & Mckiernan, 1992) and sexual violence exist, such as the abuse of Maria Cahill, which she states was perpetrated and covered up by PIRA (Cahill, 2024), or the case of Lorraine McCausland and others who were victims of the UDA Romper Rooms, where they were gang-raped and murdered (Ryan, 2023), such documentation is rare. Other incidents include women experiencing harassment by British soldiers, being assaulted by interrogators (Harris & Healy, 2001), or enduring sexualised and gendered abuse and punishment in prison (Scraton, 2020). However, these cases are often portrayed as isolated instances of sexualised violence rather than being directly linked to the conflict, despite the conflict itself providing motives, opportunity, and impunity for such acts.

Similarly, while there is a large body of feminist and critical research that highlight strip searching as a sexually violent act within the conflict or as state sanctioned sexual violence (Aretxaga, 1997, 2000, 2001; Corcoran, 2006; Pickering, 2002; Scraton, 2020; Wahidin, 2016; Wahidin & Powell, 2017), this has been strongly contested and has caused splits in feminist circles. The debate around what counts as CRSV has caused greater harm for victims and survivors of CRSV in NI as they and their experiences are unrecognised and unacknowledged. They receive little support and often cannot claim compensation or justice for the harm they have suffered. Moreover, within certain communities, both state and non-state actors, including those who perpetrated acts of gendered violence, are remembered as heroes of the conflict, while women’s experiences are underplayed and normalised (Ní Aoláin, 2000; Wahidin, 2016). Gilmartin (2018) emphasises that it is against the background of the conflict and the continuing struggle for peace that male dominance and power underlie the violent reality of women’s daily lives in NI.

This systematic review set out to collate first-hand accounts and experiences of women and girls of all ages who experienced CRSV between 1968 and 1998 in NI, because these have been largely overlooked in studies of the NI conflict and studies of CRSV. In addition, we sought to:

1. Identify and interpret prominent or recurring CRSV themes in relevant NI literature.
2. Synthesise the findings and conclusions of the most relevant qualitative literature on women and girls and their experiences of CSRV and its impact in NI during the period.
3. Identify gaps in the subject field regarding the experiences and impacts of CRSV on women and girls between 1968 and 1998 in NI.
4. Explore the effects of CRSV on women and girls between 1968 and 1998 in NI that were directly affected, as well as on NI society more broadly, to begin mitigating some of the harms caused.

## Methods

### Search Strategy

A review protocol was registered for this study with PROSPERO (ID CRD42024493247). To structure the search across all databases, we adopted a Sample, Phenomenon of Interest, Design, Evaluation, Research type (SPIDER) framework (Table 1) because, more than other methods, it can identify relevant qualitative and mixed-method publications (Cooke et al., 2012). In March 2024 we conducted a systematic search, using seven databases: (a) PyschInfo, EMBASE, and APA Physcarticles, via OVID; (b) PUBMED; (c) CINAHL Ultimate, Criminal justice abstracts, Medline, peace research abstracts, and women’s study international, via EBSCOHost; (d) the ProQuest social sciences premium collection; (e) the Cochrane Library; (f) Scopus; and (g) Web of Science. Each was searched from inception to March 2024. The databases were chosen based on their relevance to the topic, the quality of their results, and their use by other systematic reviews. In making the selection, we took advice from the Arts, Humanities, and Social Sciences subject librarian at Ulster University.

**Table 1.** SPIDER Table Outlining Search Terms for Systematic Review.

Sample	Phenomenon of Interest	Design	Evaluation	Research Type (see Design Type)
Women Young women Girls Females Wom* Ladies Gender NI North of Ireland Northern Ireland Ireland Belfast Derry Londonderry “Armagh goal” “Armagh jail” Maghaberry Prison	1964–1998 Conflict Troubles The Troubles War “Civil war”	Focus group Interview Observation review Literature review	Sexual violence Sexual assault Sexual abuse Sexual harassment Sexual exploitation Rape “Gender-based violence” Strip searching	Qualitative Mixed methods

*Note.* SPIDER = Sample, Phenomenon of Interest, Design, Evaluation, Research type.

The search adopted the SPIDER framework (Cooke et al., 2012; Table 1). It employed a Boolean search strategy, using truncation, wildcards, and MeSH terms as appropriate, and adjusted for each database. The SPIDER framework was designed to identify publications that explore the experiences of CRSV of women and girls between 1968 and 1998 in NI. The keywords were developed in consultation with the Ulster University librarian, based on the authors’ previous literature knowledge, and a rapid review of the same topic, and reflect the authors’ experiences of working with systematic reviews (see Burns et al., 2024; Murray et al., 2024).

### Grey Literature

We also searched grey literature—outputs outside of traditional commercial or academic publishing—identified by Google Scholar. Three search variants were submitted: (a) “Conflict-related sexual violence against women and girls during the Northern Ireland troubles”; (b) “Conflict-related gender-based violence against women and girls during the Northern Ireland troubles”; and (c) “Strip searching of women and girls during the Northern Ireland Troubles.” The first hundred sources were reviewed to ensure that they matched the research purpose. Book reviews were excluded from the results, but book titles were noted and consulted. We also searched several specialist websites, namely those of Amnesty International, the UK Ministry of Justice, the NI Department of Justice, and the OHCHR.

The search terms used in this systematic review were selected to comprehensively capture the various forms of CRSV experienced by women and girls in NI during the Troubles (1968–1998). As we outlined earlier, in line with Swaine’s (2022) definition of CRSV, this review integrates terms that reflect both overt and covert manifestations of CRSV.

Terms such as “rape,” “sexual violence,” and “gender-based violence” capture certain types of CRSV. References to “Armagh jail” and “Maghaberry prison” identify institutional settings in which some of these forms of abuse took place. Geographic and gendered markers ensure that references are relevant to NI and the Troubles, while the timeframe ensured that publications on periods before or after the Troubles have been excluded. These terms align with established methodologies in systematic reviews on gendered violence in conflict settings (Rubini et al., 2023).

### Inclusion and Exclusion Criteria

The inclusion criteria selected publications that:

(1) Referred to qualitative experiences of women and girls and the NI conflict and occurred between 1968 and 1998. Publications that referred to the experiences of people who identified as male were only included if results relating to people who identified as female could be isolated.
(2) Were qualitative or mixed method publications from which qualitative findings could be extracted.
(3) Included women and girls of all ages. This criterion recognised the pervasive nature of sexual harassment and violence in NI at this time. For the majority of victims, initial encounters and memories often occurred during their childhood or teenage years and continued throughout their lifetime.
(4) Were published in any language.
(5) Were peer-reviewed publications or grey literature, including reports, editorials, and PhD theses.

The search excluded publications that: (a) did not include the direct personal experience or testimony of women and girls that had experienced CRSV, or did not allow the experiences of people who identified as male and people who identified as female to be separated; (b) described CRSV that did not occur in NI; (c) described CRSV that occurred before or after the period 1968–1998.

The inclusion and exclusion criteria for this systematic review were shaped by feminist theory, which emphasises the gendered nature of violence and prioritises the lived experiences of women and girls (Ramazanoglu & Holland, 2002). Feminist scholars, including Kelly (1988) and Davies and True (2015), have conceptualised CRSV not merely in terms of interpersonal harm but also in terms of broader structural inequalities and patriarchal power. Guided by these principles, only qualitative or mixed method publications with extractable qualitative findings were included, enabling the experiences of women and girls affected by CRSV in NI between 1968 and 1998 to be explored in detail. This approach ensured methodological rigor but also prioritised subjective narratives, which feminist theorists argue is an essential step both to challenge dominant discourses and understand CRSV in a gender-sensitive way.

Publications were selected for their capacity to provide authentic insights into the lived realities of women and girls. Studies featuring men were included only when data specific to women and girls could be clearly isolated, reflecting a commitment to prioritise gendered analysis. By including women and girls of all ages, the review acknowledges the pervasive nature of CRSV and its long-term psychological and societal effects.

The inclusion of multilingual sources and both peer-reviewed and grey literature further reflects feminist methodological values. These aim to challenge epistemic hierarchies, recognise diverse forms of knowledge (Malina & Nutt, 2000), reduce selection bias, and give voice to underreported survivor testimonies. To maintain the review’s contextual and analytical integrity, it excluded publications that lacked direct personal testimonies, that generalised gendered experiences without differentiation, or that examined instances of CRSV outside NI or the defined timeframe.

### Study Selection and Data Extraction

We used the PRISMA (Page et al., 2020) flowchart as a guide for the review and reported the review in accordance with the “enhancing transparency in reporting the synthesis of qualitative research” (ENTREQ) statement (Tong et al., 2012). All database search results were exported to the Rayyan desktop software and duplicates removed. The lead reviewer reviewed all the search results and screened titles and abstracts against the inclusion and exclusion criteria. A second reviewer screened 20% of the results. The reviewers recorded discrepancies and resolved them through discussion. Both reviewers then independently subjected all publications that met the inclusion criteria to a full-text review. To make sure that they identified all relevant publications, they also screened the publications cited in the bibliographies of all included full-text publications against the inclusion and exclusion criteria, subjecting them to a full-text review if they met the inclusion criteria. The reviewers also monitored reviews of newly published works.

**Figure 1.**
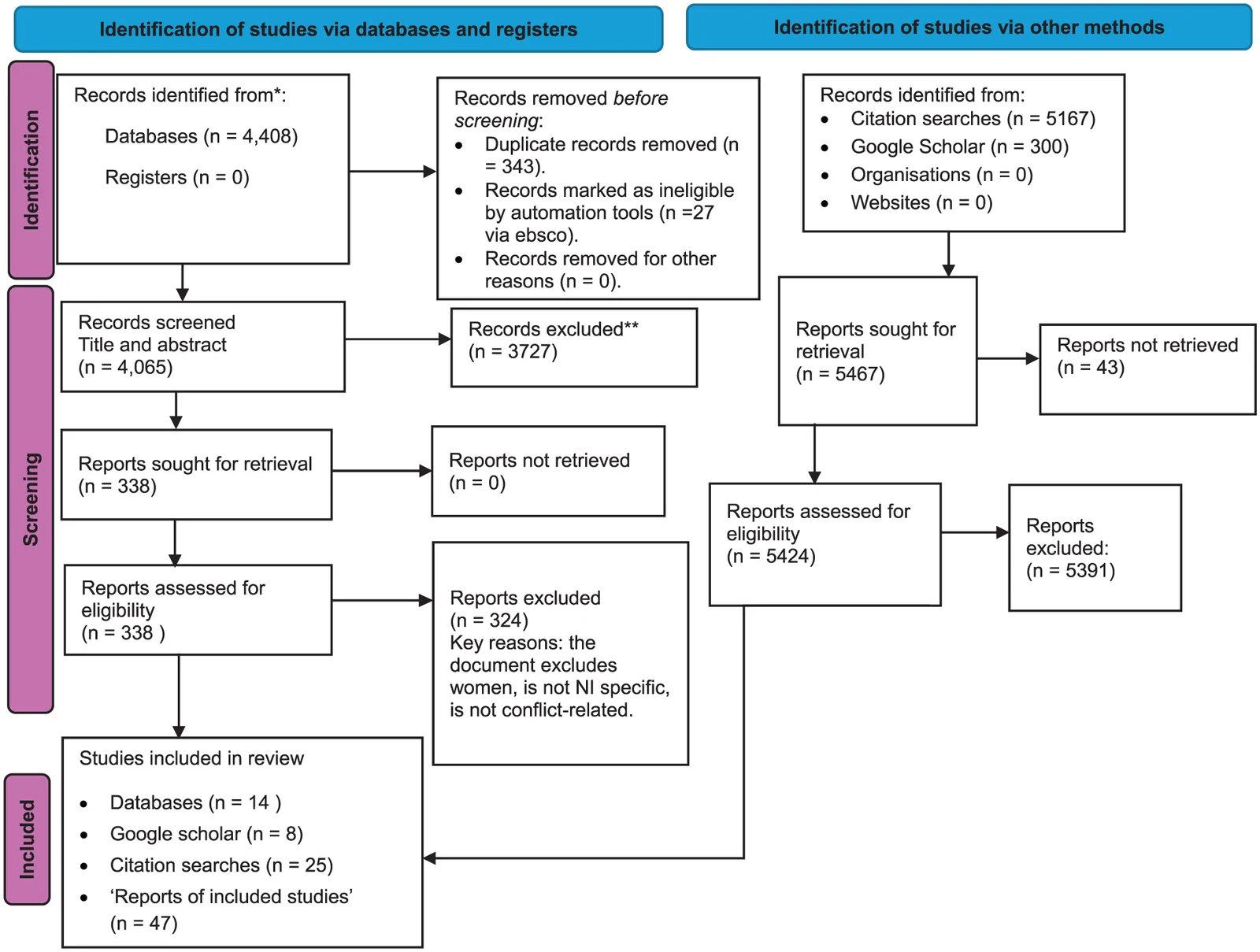
.

The results of the final search and selection process are documented in Figure 1 (PRISMA flow diagram for the original systematic reviews). The lead reviewer extracted publication data, including author(s), date published, research setting, aim(s), design, methods, sample size, sexual violence types, perpetrators, victims, locations, findings, and recommendations. The second reviewer checked the extracted data. Table 2 presents an overview of the extracted data.

**Table 2.** Data Extraction.

Source	Setting (CJ / COM) and location	Design	Paper type	Measures	ST N-ST	Victims	Findings
O’Keefe (2017), “Policing unruly women: The state and sexual violence during the Northern Irish Troubles.”	CJ and COM. During raids, patrols, in rural roads, streets, in interrogation centers, prison.	Feminist ethnographic research, including qualitative interviews and documentary and archival research. Analysed testimonies of women’s experiences with state forces.	Journal article.	Sexual assault, verbal and physical harassment, strip searches.	ST.	Republican women, political prisoners, Catholic areas, working class.	Through prison authorities, police, soldiers, and other security forces, the state weaponised sexual violence in NI as a means of disciplining and punishing republican women who were deemed deviant because they transgressed gender norms.
Gilmartin and Scull (2021), “Republican women and Catholic Church responses to the strip searching of female prisoners in Northern Ireland, 1982–1992.”	CJ .In prison.	Based on semi-structured interviews with 40 women, of whom 31 were IRA/“republican movement,” 6 were members of Sinn Féin, and 3 were “republican activists.” Most interviewees were between 50 and 70 years old.	Journal article.	Strip searches, sexual threats.	ST.	Republican women, political prisoners, prison visitors.	Strip searches and sexual violence were central features of penal life for female republican prisoners. The state deliberately used gender and sex as a weapon of war, through strip searches and other forms of sexual threats and violence. Strip searches had a particular impact on women who had conservative Catholic values. The article documented the role played by some Catholic clergy in the campaign against strip searches.
Aretxaga (2001), “The Sexual Games of the Body Politic: Fantasy and State Violence in Northern Ireland.”	CJ. In prison.	Narratives of the strip searches of 1992. Handwritten by prisoners or based on interviews with released prisoners.	Journal article.	Verbal harassment, strip searches, sexual assault.	ST.	Women prisoners.	The strip searches of 1992 were a mise en scène of military mass rape. Maghaberry women prisoners sued the prison governor for sexual harassment. Strip searches were used to subdue women, using their Catholicism and their femininity.
Swaine (2018), “Conflict-related violence against women: Transforming transition.”	COM .At home, at checkpoints, in community.	Based on semi-structured interviews with service providers who were working directly with victims of violence.	Book.	Verbal and physical harassment, sexual assault, rape.	ST and N-ST.	Women in NI.	The conflict created opportunities for violence and simultaneously lowered sanctions for perpetrators. Both state and non-state actors enjoyed impunity. Because crimes were not reported, acts of violence were not identified and were not linked to the conflict.
Corcoran (2006), “Out of order: The political imprisonment of women in Northern Ireland, 1972–1998.”	CJ .In prison.	Based on interviews with fourteen former women prisoners, of whom 12 were republican, one was loyalist, and one was non-political.	Book.	Strip searches, sexual assault, verbal harassment.	ST.	Republican and political prisoners.	In NI, republican female prisoners felt that the state, sexual violence, penal power and the bodies of confined women coincided. Threats of sexual assault and degrading comments were used to try to break women.
Van Ooijen et al., (2023), “Speaking Out on Sexualized Violence Through Artistic Storytelling in Post-conflict Northern Ireland.”	COM. In own home, in safehouse in the Republic of Ireland.	Based on interviews with 12 participants in Theatre of Witness productions. They included artistic directors and facilitators of theatre projects, and researchers from Ulster University in Derry-Londonderry. Follow-up interviews were conducted with five women who participated in the theatre projects.	Journal article.	Sexual abuse, verbal harassment.	ST and N-ST.	Women combatants and civilians.	Within the patriarchal structures of Northern Irish society, sexualised violence was not discussed. This silence was enforced by deep-seated divisions between the ethno/nationalist/sectarian communities in conflict.
Wahidin and Powell (2017), “‘The Irish Conflict’ and the experiences of female ex-combatants in the Irish Republican Army.”	CJ. In prison.	Based on semi-structured interviews with 28 women.	Journal article.	Strip searches.	ST.	Women prisoners, Women combatants.	Prison experience was marked specifically by assaults on the femininity of women, who were sexually modest as a result of their socialisation and NI’s ethno–nationalist iconography of femininity.
Harris and Healy (2001) “‘Strong about it all. . .’ rural and urban women’s experiences of the security forces in Northern Ireland.”	COM. In own home, in safehouse in the Republic of Ireland.	Stories/narratives based on interviews with 28 women from the Castlederg and Derry area.	Book.	Strip searches, verbal and physical harassment.	ST.	Nationalist women.	The security forces imposed an armed patriarchy on women in the North, who often received little support from their community and were ignored by officials. The book describes the everyday life of women.
McVeigh (1994), “‘It’s Part of Life Here....’: Security Forces and Harassment in Northern Ireland—Based on a Survey of Young People.”	COM .On the street, in communities, at stops, during patrols.	Based on a quantitative postal questionnaire followed by additional qualitative analysis in the form of a further questionnaire and approximately thirty interviews.	Research paper.	Verbal and physical harassment.	ST.	Women in NI.	Civilians but also policewomen were verbally harassed. Policing was heavily masculine and, for political as well as religious reasons, gender was a point of vulnerability for women.
Wahidin (2016), “Ex-combatants, gender and peace in Northern Ireland: Women, political protest and the prison experience.”	CJ .In prison.	Based on interviews with 28 women from across Ireland. Some had spent time in prisons in the United Kingdom, others in prisons in the Republic of Ireland or the North of Ireland.	Book.	Strip searches, verbal harassment, sexual assault.	ST.	Women prisoners.	Strip searches were so frequent that women normalised them. Republican women were particularly targeted. Searches were ostensibly “for security,” but rarely found anything and were carried out in a sexualised way.
Pickering (2008), “Women, the home and resistance in Northern Ireland.”	COM .Own home, during raids.	Based on interviews with 100 women from republican, nationalist, unionist, and loyalist traditions.	Journal article.	Verbal and physical harassment.	ST.	All women, most republican.	The security forces often used women’s sexuality as a weapon to subjugate, humiliate and degrade them while their homes were being raided. Gender specific threats and intimidation on the streets acutely shaped women’s experience. Despite this, many republican women desexualised their experiences.
Aretxaga (1997), “Shattering Silence: Women, Nationalism, and Political Subjectivity in Northern Ireland.”	CJ and COM .In prison, in community.	Based on interviews with Catholic women in west Belfast aged between 20 and about 50.	Book.	Strip searches, verbal harassment, sexual assault.	ST.	Women prisoners.	Forms of institutional and sexual domination were frequently used, especially in prisons, to exert power and control and elicit confessions. Women’s struggles were often ignored and minimised by their communities as well as by the state and courts. The female body was often colonised.
Campbell and Connolly (2007), “Deadly Complexity: Law, Social Movements and Political Violence.”	CJ .During raids, at checkpoints, in interrogations.	Based on semi-structured interviews with activists incarcerated for IRA-linked activities, including 3 women.	Journal article.	Verbal and physical harassment, sexual assault.	ST.	Republican women.	Unlike men, female encounters with the state tended to have an implicit or explicit sexual character, especially in prison where threats of sexual violence were common.
Fairweather et al., (1984), “Only the Rivers Run Free—Northern Ireland: The Women’s War.”	CJ and COM .In prison, in own home, in community.	Based on interviews with Catholic and Protestant women prisoners in Armagh.	Book.	Strip searches, rape, verbal harassment.	ST and N-ST.	Women in NI.	Men associated with paramilitaries were protected from accusations of sexual assault. Soldiers who patrolled the ports made sexual comments about women traveling away for abortions. Women were drawn in army cartoons with sexual captions (the “rapable terrorist”). Women led reclaim the night marches. In a conservative society influenced by Catholic morality, prison authorities and paramilitary interrogators both used sexual comments to break women. The UDA allegedly owned brothels. Women were “romper roomed” (tortured or killed) for going with the “wrong man.” There were allegations of rape during a religiously mixed party.
Green (2019), “The impact of conflict on violence against women in Belfast.”	COM. In prison, in own home, in community.	Based on interviews with politically aligned and non-aligned members of both communities.	Working paper.	Strip searches, tarring and feathering, rape.	ST and N-ST.	Women in NI.	Both the state and paramilitaries used sexuality and gender to control and intimidate women. The conflict exposed women to added violence because they were less able or willing to report it.
Brady et al.. (2011), “In the footsteps of Anne: Stories of republican women ex-prisoners.”	CJ .In prison.	Based on interviews with republican prisoners.	Book.	Strip searches, verbal and physical harassment.	ST.	Republican women prisoners.	These testimonies mainly describe the physical violence, bruising, and pain that strip searches caused. But women also touch on their humiliation and stress (loss of periods, crying, shaking, fainting). The book discusses the impact of strip searches on women's songs and poetry.
O’Keefe (2013), “Feminist Identity Development and Activism in Revolutionary Movements.”	CJ and COM. In prison, in own home, at IRA events.	Based on semi-structured interviews with republican women, including many combatants.	Book.	Strip searches, verbal and physical harassment, sexual assault, rape.	ST and N-ST.	Women prisoners, women combatants, republican women.	This book discusses the sexualisation of female combatants, who were called promiscuous; the attitudes of republican prisoners, who considered strip searches to be sexual assault and rape; and paramilitary control over women through tarring and feathering.
McCafferty (1981), “The Armagh Women.”	CJ and COM. In prison, in community.	Based on interviews with working class women from Catholic ghettos.	Book.	Sexual assault, verbal and physical harassment, strip searches.	ST and N-ST.	Catholic women, prisoners.	Sexuality and gender were used to oppress women both by men in their communities and by the state during the conflict.
Gallagher (1992), “Troubles and Joy: An Anthology of Craigavon Women Writers.”	COM. Interrogation and prison.	Short stories and poetry by 40 women from Craigavon.	Book.	Strip searches.	ST.	Prisoners.	These poems highlight the impact of strip searches on women and compared them to sexual assault and rape.
Pickering and Third (2003), “Castrating Conflict: Gender(ed) terrorists and terrorism domesticated.”	CJ and COM. In house raids, in interrogation.	A qualitative study based on interviews with 100 politically active women in Northern Ireland.	Journal article.	Verbal and sexual abuse.	ST.	Politically active women.	The article highlights that women’s bodies were sites of oppression and resistance, not least because of traditional gender roles.
Corcoran (2003), “‘Doing Your Time Right’: The Punishment and Resistance of Women Political Prisoners in Northern Ireland, 1972–1995.”	CJ. In prison.	Based on semi-structured interviews with 14 former prisoners who had been held in Armagh or Maghaberry prisons between 1972 and 1995.	PhD thesis.	Verbal and sexual abuse, strip searches, body searches.	ST.	Women prisoners.	The thesis shows that women in Northern Ireland suffered verbal and sexualised violence while in prison. Their experiences were frequently gendered, and punishments targeted gender and sexuality specifically. Strip searching was perceived as sexual violence.
Mairs (2011), “Unseen Women.”	CJ .In prison, in interrogation.	Based on recordings made by three republicans, two prison officers, and one loyalist.	Video and book.	Strip searches, verbal sexualised abuse.	ST.	Women prisoners.	State actors used threats during interrogation. Strip searches were employed to embarrass, humiliate, and degrade women.
Gallagher (1997), “Women Imprisoned as a Result of the Struggle.”	CJ. In prison.	Based on three interviews with republican prisoners.	Journal article.	Strip searches.	ST.	Women prisoners.	Strip searches were used as a psychological weapon, which prisoners considered akin to rape.
Moore (2014), “Women in ethnic conflict: a critical ethnography of female combatants in the Provisional Irish Republican Army.”	CJ and COM .In prison, in interrogation, in street.	Based on interviews with one Protestant probation officer during the time of Armagh Gaol protests, two political/community republican activists, eight ex-IRA, ex-prisoners, and current Sinn Féin members.	PhD thesis.	Physical and sexual abuse, strip searches.	ST.	Republican women and prisoners.	The thesis argues that during the Troubles the authorities used gendered violence systematically against female republican prisoners in Armagh Gaol. They targeted women’s sexuality and religion.
Wahidin (2018), “Menstruation as a Weapon of War: The Politics of the Bleeding Body for Women on Political Protest at Armagh Prison, Northern Ireland.”	CJ .In prison.	Based on 28 qualitative interviews with women who had experienced imprisonment and who participated in the No Wash protest in Armagh prison during the 1980s.	Journal article.	Strip searches.	ST.	Women prisoners.	The article focuses on menstruation, the “desexing” of female prisoners, and the feminisation of officers. Interviewees described strip searches as sexual assaults, said that they feared internal injury, and reported that male interrogators participated if they struggled.
Mukungu (2022), “Women’s anti-violence activism relations in post-conflict Namibia and Northern Ireland.”	COM .In community.	Twenty anti-VAWG activists participated in this study.	PhD thesis.	Sexual and domestic violence, rape.	N-ST.	Women in NI.	This thesis did not find that Sexual violence or GBV was used strategically by paramilitary groups against women from other communities, but reported that it did occur. It found that state armed forces used force to intimidate women in working class republican communities, and punish republican women who were thought to be involved in subversive anti-state or paramilitary activity.
Aretxaga (2000), “Engendering Violence: Strip Searches of Women in Northern Ireland.”	CJ .In prison.	Based on open-ended interviews with imprisoned women who had experience of strip searches.	Journal article.	Strip searches, sexualised remarks.	ST.	Women prisoners.	This article concluded that the strip search of 1992 followed a consistent pattern of militarised sexualised assault. It targeted the women, but also the community of prisoners, the nationalist community, and the Irish nation.
The London Armagh Group (1984), “Women behind the wire.”	CJ .In prison, in interrogation.	Interviews and women’s own words.	Pamphlet.	Sexual harassment, verbal threats, strip searches.	ST.	Women prisoners.	The pamphlet reports that soldiers forced young women to perform sexual acts on themselves at gunpoint, that a woman was verbally harassed and threatened during interrogation, and that strip searches were used to harass women.
Calamati (2002), “Women’s Stories from the North of Ireland.”	CJ .In prison, in interrogation.	Testimony and interviews.	Book.	Sexual harassment, physical touching, verbal abuse.	ST.	Republican women.	Women discuss how male (and female) interrogators used verbal and physical sexual violence against them. Threats of rape were often accompanied by inappropriate kissing or touching.
Campbell and Connolly (2006), “Making War on Terror? Global Lessons from Northern Ireland.”	COM. In community.	Based on semi-structured interviews with activists incarcerated for IRA-linked activities, including 3 women.	Journal article.	Verbal harassment.	ST.	Women.	The article reports that state actors verbally harassed women on a gendered basis.
Pickering (2000), “Engendering Resistance: Women, and Policing in Northern Ireland.”	CJ .During interrogation.	Based on in-depth interviews with 100 politically active women.	Journal article.	Sexualised comments.	ST.	Women in NI.	The article reports that the security forces used explicitly gendered and sexualised forms of interrogation, putting gender at the center of the struggle for political identity and bodily integrity.
Pickering (2002), “Women policing and resistance in Northern Ireland.”	CJ and COM .In community, in interrogation.	Based on in-depth interviews with 100 politically active women.	Book.	Sexualised comments and threats, inappropriate touching, sexual assault, strip searches.	ST.	Women in NI.	The book notes that the security forces exploited gender in their daily contacts with women in NI and that this marked women’s experiences of “formal” contact with the state.
Faul and Murray (1978), “The Castlereagh File: Allegations of RUC Brutality 1976–1977.”	CJ .In interrogation.	Testimonies and interviews.	Pamphlet.	Verbal sexualised harassment, physical abuse.	ST.	Women in interrogation.	The pamphlet argues that sexualised and gendered ill-treatment of girls and women was widespread, occurred in a variety of interrogation centers, and affected girls from the age of 13 and women of a variety of ages.
McAuley (1989), “Women in a war zone: 20 years of resistance.”	CJ .In-house raids, during arrest, in interrogation.	Based on interviews with IRA volunteers, Sinn Fein political prisoners, and families.	Book.	Sexualised comments, physical harassment, strip searches.	ST.	Republican women and prisoners.	Interviewees report that women were subject to sexual abuse. If they complained they were threatened with further harassment or sexual assault. The interviewees say that strip searches had the same motives as rape and inspired the same feelings in women.
Shannon (1997), “I am of Ireland.”	CJ and COM .In home, in prison, in interrogation.	Based on testimonies and interviews.	Book.	Physical harassment of a sexualised nature, strip searches, verbal harassment.	ST and N-ST.	Prisoners and wives of nationalist politicians.	The book includes the testimony of Anita Currie, who was attacked by a loyalist gang who carved UVF into her breast; and also the testimonies of loyalist women, who say that interrogators used verbal sexualised comments during questioning. The book states that strip searches were considered a psychological weapon.
MacDonald (1991), “Shoot the women first.”	COM .In community.	Based on interviews.	Book.	Verbal harassment.	ST.	Ex-prisoners.	The book notes that soldiers and RUC officers verbally harassed women.
Fitzpatrick (2015), “Gender and Affect in Testimonial Performance: The example of I Once Knew a Girl.”	COM .In own home, in IRA missions.	Based on interviews with six women from various backgrounds.	Journal article.	Sexual assault, rape, domestic abuse.	N-ST.	IRA quartermaster and family.	These interviewees highlight the systemic nature of violence against women during conflict, and point out that sexual violence or GBV is too often assumed to be a lesser form of violence.
Belfrage (1987), “The Crack: A Belfast Year.”	COM .In community, in home.	Based on interviews.	Book.	Verbal threats, assault.	N-ST.	Family members of paramilitaries.	The book notes that the families of paramilitaries were exposed to gendered and sexualised threats from other paramilitaries.
Gallagher (1995), “An Oral History of the Imprisoned Female Irish Republic Army.”	CJ. In prison.	The life histories of eight women.	PhD thesis.	Strip searches.	ST.	Women prisoners.	This thesis argues that women in Armagh believed that strip searches were a form of psychological rape, used as a weapon by both female and male prison guards.
Christian Response to Strip Search Group (1997), “‘Christian Study: A Community Problem,’ A Christian Response to Strip Searching.”	CJ. In prison.	The testimonies of two women.	Pamphlet.	Strip searches.	ST.	Women prisoners.	Women who experienced strip searches believed they were a form of legitimised rape.
Belfast Women’s Collective, “Scarlet Women.”	CJ .In prison.	One letter from prison.	Journal article.	Harassment, verbal abuse.	ST.	Female political prisoner.	Women felt deeply afraid and vulnerable while in prison, particularly in the presence of male prison officers.
London Strategic Policy Unit (1988), “Working Together to End Strip Searching: Report of a Conference.”	CJ .In prison.	The testimony of a 17-year-old woman in Armagh.	Conference report.	Strip searches.	ST.	Woman prisoner.	The interviewee says that, in an effort to “defeat her,” officials used strip searches to control her but also to force her to confront her gender, sexuality and vulnerability.
Shanahan (1988), “Waiting for Justice? One Woman’s Story.”	CJ. In prison.	A personal testimony.	Pamphlet.	Strip searches.	ST.	Woman prisoner.	Women who were strip searched say that it was a humiliating and degrading practice, used to harass women rather than improve security.
Community for Justice (1992), “Strip Searching: A Moral Issue.”	CJ. In prison.	An article.	Magazine.	Strip searches, verbal harassment, physical harassment.	ST.	Women prisoners.	Strip searches caused feelings of degradation and revulsion.
Women in Struggle (1992a), “Women in Struggle Volume 2.”	CJ .In prison.	An article.	Magazine.	Strip searches, verbal harassment, physical harassment.	ST.	Women prisoners.	The article emphasises the intensity of women’s feelings: both rape and strip searches were acts of violence to gain power and control over women.
Women in Struggle (1992b), “Women in Struggle Volume 4.”	CJ .In prison.	One interview with a woman.	Magazine.	Strip searches, verbal harassment, physical harassment.	ST.	Political prisoner.	The article highlights that women prisoners felt that prison officers used strip searches to manipulate women’s sexuality against them.
Women Against Imperialism (1980), “Women Protest for Political Status in Armagh Gaol.”	CJ .In interrogation, in prison.	The testimonies of four interrogated women.	Report.	Strip searches, verbal harassment, physical harassment.	ST.	Women prisoners and interrogations.	The report found that, during interrogations, women were verbally and physically harassed during strip searches and questioning.

*Note.* CJ = Criminal Justice; COM = Community; NI = Northern Ireland; N-ST = Non-state Perpetrator; ST = State Perpetrator.

### Quality Assessment of Individual Publications

The research team used the Critical Appraisal Skills Programme (CASP) (CASP-UK, 2018a, 2018b), to assess the validity and integrity of the qualitative publications’ methodology, findings, and impact. The two reviewers independently appraised all included publications against a 10-question checklist and recorded a “yes (shaded in green),” “no (shaded in red),” or “can’t tell (shaded in yellow)” response to each question. Table 3 displays the outcome. Between 1 and 3 “yeses” was a low-quality result; between 3 and 6 “yeses” was a medium-quality result (shaded in pink); and between 7 and 10 “yeses” was a high-quality result (shaded in purple).

**Table 3.** CASP Quality Assessment—Summary Table.

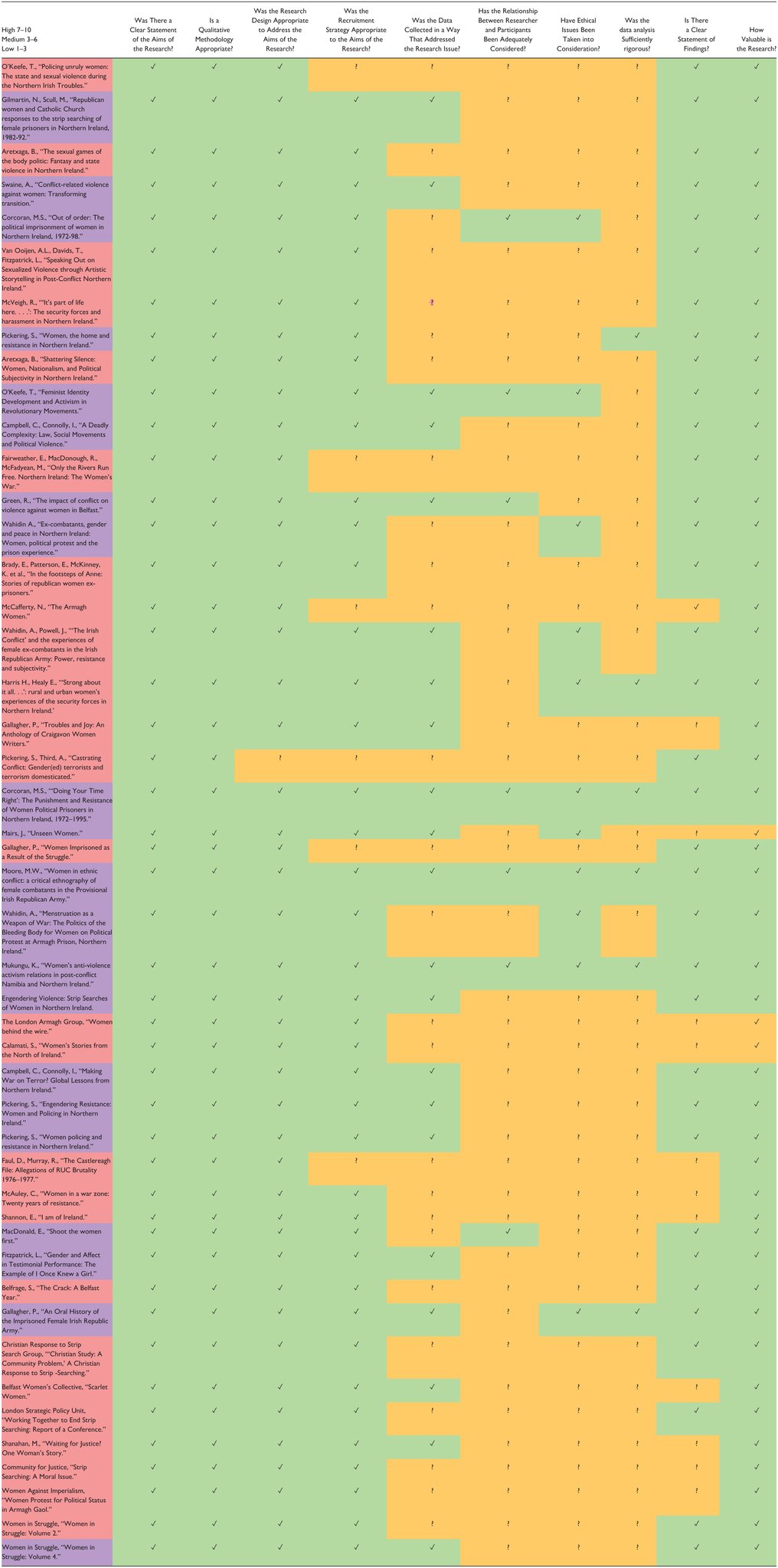

*Note*. CASP = Critical Appraisal Skills Programme.

### Data Synthesis

Because the inclusion criteria largely identified qualitative publications, the research team undertook a thematic analysis (Braun & Clarke, 2006). Thematic analysis is the most appropriate methodology for qualitative publications (Braun & Clarke, 2006; Bryman, 2012) because it can sustain an explicit and transparent link between the texts of primary publications, their results, and discussions—a key principle of systematic reviews (Thomas & Harden, 2008). The two reviewers read through and analysed every included source, coded it line-by-line, and selected key themes that emerged. The initial codes were then re-examined and condensed into smaller, conceptually similar “descriptive themes.” In the final stage, they interpreted the descriptive themes and translated them into analytical themes that met the aims of the review.

## Strengths and Limitations

This review employed a rigorous and transparent methodology to ensure validity, reliability, and credibility. The use of the SPIDER framework and a systematic search across multiple databases enhanced the identification of relevant qualitative publications, while dual-reviewer screening and thematic analysis minimised bias and strengthened consistency in data interpretation (Cooke et al., 2012). Adherence to PRISMA guidelines, ENTREQ reporting, and PROSPERO registration further reinforced transparency and methodological clarity (Page et al., 2020). Nonetheless, some limitations remain. The interpretative nature of thematic analysis introduces the possibility of subjective bias, and the predefined inclusion criteria may inadvertently have excluded publications that offer alternative perspectives, as discussed below. Furthermore, while grey literature broadens the scope, inconsistencies in quality across non-peer-reviewed sources may have impacted overall reliability. These considerations are acknowledged to provide a balanced evaluation of the reviews approach while maintaining its methodological integrity.

This review has located, appraised, and synthesised primary qualitative data on CRSV that women and girls experienced during the Troubles. The evidence draws on 47 publications, which may seem small for a systematic review. However, the small geographical location of NI as well as the lacuna of research on CRSV in both research and policy makes the review robust in size. Although some of the publications were not written to academic standards or for an academic audience, their quality was ranked as high or medium due to the validity and relevance of the research. All of the 47 publications included first-hand experiences of women and girls as described in their own words.

The review focused on capturing the first-hand experiences of women and girls. It did not include second-hand accounts, may have missed some direct testimonies and reports, and did not pick up the experiences of women and girls who chose to remain silent. As the findings highlight, the experiences and voices of PUL women and girls are missing from the vast majority of narratives about the conflict, partly because these people are not easy to access, and some are unwilling to speak out. Further research is needed to fill this gap.

Because the review focused on the experiences of women and girls, it missed accounts of CRSV experienced by people who identified as male. It would be wrong to conclude that CRSV was not experienced by people who identified as male in NI during the Troubles, though the motivations for it, the experience of it, and the feelings and effects differ between genders. More research is needed to gain a full and accurate picture of CRSV in NI generally.

This review chose to exclude search terms relating to historical institutional and clerical abuse. Although studies have shown that the conflict created conditions in which such abuse could occur (McCormick et al., 2021; Moore, 1996), such abuses are not limited to the period of the Troubles. Including them would provide insights into a small fraction of the abuses that occurred and would not supply the broader historical and social context, which is necessary to fully understand their scale and significance.

## Results

In total, 4,408 records were collected from the 12 databases, and 343 duplicates were removed. At sift one, title and abstract searches were conducted on the 4,065 remaining publications to check the relevance of results. At sift two, 338 of these were subjected to full text review, of which 324 were excluded. Fourteen publications were finally included from the database searches.

After reviewing citations and grey literature, a further 33 publications were included in the study. We initially conducted a backward citation search by reviewing the bibliographies of the 14 publications identified through database searches, which yielded an additional 25 relevant sources. We then performed a forward citation search using Google Scholar, examining works that had cited the total of 39 publications, and identified a further 8 relevant publications.

Altogether 47 publications (out of 9,895 results) reported the CRSV experiences of women and girls in NI between 1968 and 1998: these were included in the review. See Figure 1 for the PRISMA flow diagram of included publications.

These 47 publications collectively highlight that CRSV was not an isolated occurrence but a deeply ingrained reality shaping the lives of women and girls in NI between 1968 and 1998. The breadth of sources reinforces the persuasive nature of this violence across both community settings and state institutions. While certain publications focus on specific incidents, such as the mass strip searches of republican prisoners, the overarching pattern points to CRSV as a clear mechanism of control and intimidation.

### Characteristics of the Included Publications

Table 2 presents the data extracted from the included publications. Reflecting the review’s focus, almost all the publications and associated data were written or gathered in NI or the Republic of Ireland. Similarly, because it searched for the direct experiences of women and girls, all the selected publications featured the experiences of women and girls. Across all publications, the experiences documented were exclusively those of cisgender women and girls (assigned female at birth). Consistent with the review’s focus, they all contained a qualitative component: all 47 publications included interviews, or contained testaments written by victims, in the form of direct quotations, as required by the inclusion criteria. Only one included publication employed a mixed-method approach.

Nineteen of the included publications were books; 13 were academic journal articles; 6 were pamphlets; 4 were PhD theses; 3 were in non-academic journals or magazines; 1 was a working paper; and 1 was a conference report. Thirty-six of the publications were published between 1981 and 2013, while 11 were published in the last 10 years. They tended to examine a criminal justice setting (26 publications), a community setting (12 publications), or a criminal justice and community setting (9 publications).

All 47 publications referred to the CRSV experiences of women and girls. Nine focused specifically on the experiences of Catholic women and girls; six on the experiences of female combatants in their role as combatants (usually from republican movements); and 31 on the experiences of female republican political prisoners or on prisoners in general. Only seven explicitly included PUL experiences. Age was not always mentioned, but where it was, the participants were aged between 16 and 72 at the time of interview or testimony.

CRSV against women and girls was perpetrated by state actors, including police, prison officials and soldiers, and non-state actors, including members of loyalist and republican paramilitary organisations. Of the included publications, 37 described CRSV by state actors, 7 described CSRV by state and non-state actors, and 3 described CRSV by non-state actors only.

Many of the included publications do not say exactly where instances of CRSV occurred, particularly when they took place in a community setting. This may reflect a desire to preserve the anonymity of research participants. Nevertheless, several publications did identify locations: 25 mentioned instances of CRSV in a criminal justice context, during arrests, interrogations or imprisonment; 9 mentioned instances of CRSV in the community, for example, in the home, local area, at police checkpoints, or during raids; and 13 included instances in both criminal justice and community settings.

### Results of Quality Appraisal

Using the CASP qualitative checklist, the first and second reviewers ran a quality assessment and risk of bias assessment of all 47 included publications, to assess the results, their validity, and their potential local impact. Reviewers found that 22 were of high quality, and the remaining 25 of medium quality. It must be noted, however, that many of the sources drew on personal testimony and were not written for an academic audience, and that as a result their objectivity may vary. Additionally, all 47 included publications contained data that would have an impact locally. A full summary of questions and results is shown in Table 3.

## Data Synthesis

Four analytical themes emerged when undertaking thematic synthesis: (a) sexual violence by state actors; (b) sexual violence by paramilitaries; (c) sexualisation and victimisation of female combatants; and (d) impacts on women, girls and the wider community. Table 4 shows how the 47 selected publications addressed these themes.

Each theme is summarised in the subsections below, with examples of verbatim quotations.

**Table 4.** Themes.

Analytical theme	Descriptive theme	Number	Example quotes from text	Paper
Harassment by state actors	• Verbal, physical, sexual, and psychological violence during arrest and interrogation.	23	“They would mention the name of a man and say “Did you fuck him?” and continue, “You Catholic girls just fuck anybody, you sluts. You know we can rape you right here, and nobody is going to believe you, because you are all fucking taigs.”. . .” [About her breasts] “Who gropes them? Everybody in the IRA has gotten their hands on them at some point. . . . We know all about you: you all have your legs permanently opened for the cause and for Ireland.”	Aretxaga (1997)Page 113
	• Strip searches in prisons.	32	“They then proceeded to bring in more officers to hold you down while they [the prison officers] took your clothing off and when you were completely naked you were then bent over and they would do an internal search of your anus and vagina, and all the while you're struggling and struggling.”	Wahidin and Powell (2017)Page 562
	• Verbal, physical, sexual, and psychological violence during house raids.	13	“Who did you service last night?,” you know. Or looking at your underwear, again derogatory remarks about my underwear, “Who were you wearing this for? What bastard were you wearing this for last night?”	Harris and Healy (2001)Page 54
	• Verbal, physical, sexual, and psychological violence in the community.	17	“The Grenadier Guards [a British army regiment] gave us a really hard time. Especially, verbal, sexual, every type of harassment that they could, they have a reputation for that, and there wasn’t a day that there wasn’t an incident involving a young woman. They were always wanting to search them.”	Harris and Healy (2001)Page 86
Harassment by paramilitaries	• Sexual violence weaponised against members of the “other” community.	3	“They ripped open my housecoat and carved ‘UVF’ across my breast with a knife.”	Shannon (1997)Page 67
	• Policing of women’s sexuality in communities.	7	“She was ‘feathered’ for allegedly going out with British soldiers and accused of touting.”	Fairweather et al. (1984)Page 246
	• Surveillance.	5	“The Troubles had a big impact on women. . .. I know for a fact that you still had men being controlling in the jail with their wives . . . There was no normal life for the women.”	Mukungu (2022)Page 146
Sexualisation and victimisation of female combatants	• Sexualisation of the female terrorist.	1	“Female combatants as overly sexualised with captions talking about the ‘rapable terrorist.’”	Fairweather et al. (1984)Page 54
	• Presumption of promiscuity	2	“Membership of IRA resulted in other IRA members making presumptions about her promiscuity, assuming she would ‘accompany him to his hotel room for the night.’”	O’Keefe (2013)Page 89
	• Sexual violence perpetrated because of or resulting from a woman’s role in the conflict.	6	“Her commander ‘came on very strong’ and she ‘wanted to say no but didn’t know how.’”	Fitzpatrick (2015)Page 113
Impact on women and the wider community	• Constant threat of attack and knowledge of vulnerability.	31	“Just the sense of physical attack and I think the possibility of physical sexual attack makes women on their own very vulnerable, and very vulnerable too, in a situation when the police are never on their own.”	Pickering (2002)Page 151
	• Feelings during and after sexual assaults	42	“Disgust, pain, embarrassment, grief, anger, frustration.”	Van Ooijen et al. (2023)Page 1059
	• Impunity and lack of support	5	“[T]here have been cover-ups of rapes. . . They do the pretence of saying ‘we are the protectors of the community; we will investigate this’ and then take a young woman out of her house every night for a year and question her and try to get her to take back her story”	Swaine (2018)Page 113–114
	• Normalisation of violence	5	“The experiences I have had are an everyday part of life for people.”	McVeigh (1994)Page 171

### Sexual Violence by the RUC and British Army

Sexual violence by state actors was the theme the reviewed publications mentioned most frequently: it appeared in 44 of the 47. In particular, Gilmartin and Scull (2021) emphasised that, during the Troubles, women’s and girls’ bodies were used as a battleground in the criminal justice system and in their contact with state forces. Four descriptive themes of sexual violence by the RUC and British Army emerged: (a) sexual violence during arrest and interrogation; (b) strip searches in prisons; (c) sexual violence by the RUC and British Army during house raids; and (d) sexual violence by the RUC and British Army in the community. See Table 5.

**Table 5.** Sexual Violence by State Actors.

Theme	References
Sexual violence by the RUC and British Army	(Aretxaga, 1997, 2000, 2001; Belfast Women’s Collective, 1980; Brady et al., 2011; Calamati, 2002; Campbell & Connolly, 2006, 2007; Christian Response to Strip Search Group, 1997; Community for Justice, 1987; Corcoran, 2003, 2006; Fairweather et al., 1984; Faul & Murray, 1978; Gallagher, 1992, 1995, 1997; Gilmartin & Scull, 2022; Green, 2019; Harris & Healy, 2001; London Strategic Policy Unit, 1988; Mairs, 2011; McAuley, 1989; McCafferty, 1981; McDonald, 1991; McVeigh, 1994; Moore, 2014; O’Keefe, 2013, 2017; Pickering, 2000, 2002, 2008; Pickering & Third, 2003; Shanahan, 1988; Shannon, 1997; Swaine, 2018; The London Armagh Group, 1984; Van Ooijen et al., 2023; Wahidin, 2018; Wahidin & Powell, 2017; Women Against Imperialism, 1980; Women in Struggle, 1992a, 1992b)
Subthemes	References
Sexual violence during arrest and interrogation	(Aretxaga, 1997; Brady et al., 2011; Calamati, 2002; Campbell & Connolly, 2007; Christian Response to Strip Search Group, 1997; Community for Justice, 1987; Fairweather et al., 1984; Faul & Murray, 1978; Harris & Healy, 2001; Mairs, 2011; McAuley, 1989; McCafferty, 1981; McVeigh, 1994; Moore, 2014; O’Keefe, 2013, 2017; Pickering and Third, 2003; Pickering, 2000, 2002; Shannon, 1997; The London Armagh Group, 1984; Women Against Imperialism, 1980; Women in Struggle, 1992b)
Strip searches in prisons	(Aretxaga, 1997, 2000, 2001; Brady et al., 2011; Christian Response to Strip Search Group, 1997; Community for Justice, 1987; Corcoran, 2003, 2006; Fairweather et al., 1984; Gallagher, 1992, 1995, 1997; Gilmartin & Scull, 2022; Green, 2019; Harris & Healy, 2001; London Strategic Policy Unit, 1988; Mairs, 2011; McAuley, 1989; McCafferty, 1981; Moore, 2014; O’Keefe, 2013, 2017; Pickering, 2002; Shanahan, 1988; Shannon, 1997; The London Armagh Group, 1984; Wahidin, 2016, 2018; Wahidin and Powell, 2017; Women Against Imperialism, 1980; Women in Struggle, 1992a, 1992b)
Sexual violence by the RUC and British Army during house raids	(Campbell & Connolly, 2007; Fairweather et al., 1984; Green, 2019; Harris & Healy, 2001; McAuley, 1989; McCafferty, 1981; O’Keefe, 2013; Pickering, 2002, 2008; Pickering & Third, 2003; Shannon, 1997; Swaine, 2018; Van Ooijen et al., 2023)
Sexual violence by the RUC and British Army in the community	(Aretxaga, 1997; Campbell and Connolly, 2006, 2007; Fairweather et al., 1984; Green, 2019; Harris and Healy, 2001; McAuley,1989; McCafferty, 1981; McDonald, 1991; McVeigh, 1994; Moore, 2014; O’Keefe, 2013; O’Keefe, 2017; Pickering and Third, 2003; Pickering, 2002, 2008; Swaine, 2018)

Strip searches in prisons were mentioned most often: 32 of the included publications referred to them. While strip searching was an official policy, many women and girls felt it was a form of sexual violence, used by the state to control republican women and girls, their bodies and their sexuality, break their spirit, and eventually break the spirit of the republican movement.

So strip searching was to break, as they seen it, these Catholic women and they'll find this strip searching utterly and totally degrading, which it was, but it was designed to try and break us. (Former republican prisoner, in Gilmartin and Scull, 2022: 731-732.)

[Strip searching is] the ultimate weapon they have against us. You see a group of women who are extremely strong, highly politically motivated, highly personally motivated, and it's a way of containment, just a way to remind us that they have the power and control. (Former female republican prisoner, in Corcoran, 2006: 185.)

Women described sexual violence during arrest and interrogation in 23 of the included publications. Sexual violence took several forms. For example, sexually violent language was used to humiliate and demean women and girls as well as create a sense of fear and vulnerability. One woman spoke of being interrogated by RUC officers.

They would mention the name of a man and say “Did you fuck him?” And continue, “You Catholic girls just fuck anybody, you sluts. You know we can rape you right here, and nobody is going to believe you, because you are all fucking taigs^1^.”. . . [About her breasts] “Who gropes them? Everybody in the IRA has gotten their hands on them at some point. . . We know all about you: you all have your legs permanently opened for the cause and for Ireland.” (Aretxaga, 1997: 133.)

Another woman spoke of her experiences of being interrogated by a soldier in the British Army.

[The soldier said:] “Would you like a bit of dick. We’re going to rape you. We’re going to strip you. Nobody fucking knows you’re here. I can put you up that wall and I’m going to have you from the front. He’s going to have you from the back and we’ll call somebody else and have his dick in your mouth.” (Wahidin, 2016: 48.)

Many women and girls also reported experiences of physical sexual violence during interrogation.

[During interrogation the police officer] went to grope me. He put his hands between my legs. (O’Keefe, 2013: 37.)

[During interrogation] I was physically abused and sort of, in a way, sexually abused. This particular [Special] Branch man was putting his hands on my body, and putting his arms round me, and trying to intimidate me that way. (Campbell and Connolly, 2007: 290.)

During house raids, RUC officers and British Army soldiers used verbal and physical threats and actions to target women’s and girls’ bodies and spaces.

“Who did you service last night?” you know. Or looking at your underwear, again derogatory remarks about my underwear, “Who were you wearing this for?” “What bastard were you wearing this for last night?” (Woman talking about British Army soldiers, in Harris and Healy, 2001: 54.)

State actors used the vulnerability felt by the women and girls to police and control the communities. Women and girls felt that the state could wield power and authority even in their homes and that neither they nor their families were ever truly safe. One interviewee commented, for example:

To women it’s like being raped because they go through everything, through underwear drawers, they’re going through your clothes, your papers, your taxes, it’s just like being raped. (Pickering, 2008: 25.)

Seventeen of the included publications mentioned acts of sexual violence committed by the RUC and British Army.

The Grenadier guards (British army regiment) gave us a really hard time. Especially, verbal, sexual, every type of harassment that they could, they have a reputation for that, and there wasn’t a day that there wasn't an incident involving a young woman. They were always wanting to search them. (Woman, in Harris and Healy, 2001: 86.)

Much of the literature that discusses women and girls’ experiences in the criminal justice system focuses on CNR women and girls. This may be due to the frequency of CNR interactions with state forces, or because the experiences and voices of PUL women and girls are missing from most narratives of the conflict. The latter are not easily accessed, and in some cases are unwilling to speak out. Only one publication included an account by a PUL woman who experienced sexual violence at the hands of a state actor (Shannon, 1997). She reported an interrogation during which police officers tried to suggest that she was a stripper or entertainer for the Ulster Defence Association (UDA). “‘Did you dance for the UDA?’ they asked, trying to make it dirty” (Shannon, 1997: 153).

These narratives make clear that women and girl’s interactions with both the British army and criminal justice system were often violent and highly sexualised. Seifert’s (1994) theory on the motivations behind CRSV can be applied here, the assault of republican women and girls by security forces can be understood not only as an attempt to ‘emasculate’ the men who were unable to ‘protect’ their wives, daughters, and mothers, but also as a symbolic attack on republican culture itself, especially given its appropriation of the female figure of Mother Ireland. Moreover, the targeting and subjugation of politically active, subversive, and defiant women and girls echoes Enloe’s (2000) analysis of national security rape as a strategy to suppress perceived threats to state power.

### Sexual Violence by Members of Paramilitary Organisations

In 10 of the included publications, women reported that they had experienced sexual violence at the hands of members of paramilitary organisations. This analytical theme contained three descriptive themes: (a) policing of women’s sexuality in communities; (b) surveillance and a culture of “being watched”; and (c) sexual violence used as a weapon against members of the “other” community. See Table 6.

**Table 6.** Sexual Violence by Members of Paramilitary Organisations.

Theme	Reference
Sexual violence by members of paramilitary organisations	(Belfrage, 1987; Fairweather et al., 1984; Fitzpatrick, 2015; Green, 2019; McCafferty, 1981; Mukungu, 2022; O’Keefe, 2013; Shannon, 1997; Swaine, 2018; Van Ooijen et al., 2023)
Subtheme	Reference
Policing of women’s sexuality in communities	(Belfrage, 1987; Fairweather et al., 1984; Green, 2019; McCafferty, 1981; Mukungu, 2022; O’Keefe, 2013; Swaine, 2018)
Surveillance and a culture of “being watched”	(Belfrage, 1987; Fairweather et al., 1984; O’Keefe, 2013; McCafferty, 1981; Mukungu, 2022)
Sexual violence used as a weapon against members of the “other” community	(Belfrage, 1987; Shannon, 1997; Swaine, 2018)

In seven of the included publications, women and girls reported that paramilitary organisations policed their sexuality in their own communities. Women and girls were punished, usually in public, in a sexualised and humiliating way, for perceived transgressions. Fairweather et al. (1984: 246) recounted the story of a woman punished by the IRA for perceived sexual transgressions. “She was feathered^2^ for allegedly going out with British soldiers and accused of touting.”^3^ Another interviewee described how women were policed, and often punished, for their choice of partner:

And the issue of women being tarred and feathered, again women were victimised because of their sexuality, so, for going with soldiers, or, in the case of loyalist working class communities for going with Catholics. (Mukungu, 2022: 160.)

Five of the included publications mentioned the surveillance of women by paramilitary members within their communities. Paramilitary groups leveraged their influence and local networks to “watch” neighbourhoods, often receiving detailed reports on the activities and behaviours of residents. Surveillance could include whom a woman talked to, if she was wore makeup, or how she was dressed on a certain day.

The Troubles had a big impact on women. . . I know for a fact that you still had men being controlling in the jail with their wives. . . There was no normal life for the women. (Mukungu, 2022: 146.)

The wife of an UDR officer received threatening phone calls from the IRA:

The IRA will know that you’ve joined [the UDR]. . . then the phone calls started. . . “I’m coming up to rape you, Mrs Elliot”. (Belfrage, 1987: 62.)

In many cases, surveillance resulted in further policing, and paramilitary punishments if a transgression was deemed to have occurred. Punishments included social isolation, shaming, and tarring and feathering.

Three of the publications described the use of sexual violence as a weapon against members of the “other” community. In one instance, the wife of a nationalist politician described what happened when members of a loyalist paramilitary organisation came to her house in search of her husband, who was not at home.

They ripped open my housecoat and carved ‘UVF’ across my breasts with a knife. The only thing they didn’t do was rape me. (Shannon, 1997: 67)

Women’s testimonies show that during the Troubles women were controlled, watched, victimised, and punished, by paramilitaries from their own community or paramilitaries from the “other” community. The policing of women’s sexuality reflects global patterns of CRSV. As Seifert (1994) and Goldstein (2001) argue, CRSV is often deployed as a tool for asserting dominance and controlling populations. Within the NI context, these acts reinforced gendered power structures and maintained paramilitary control of communities. Women’s and girls’ awareness of being watched and controlled, and of the risk of punishment, had a hugely psychological impact. It affected their comfort, their ability to speak out, and their willingness to report or stop physical and sexual violence and other forms of oversight, enabling paramilitaries to maintain their control, power, and impunity. The fear of punishment created an environment in which women’s and girls’ autonomy was immensely constrained, reflecting the function of CRSV described by Yuval-Davis and Anthias (1989), wherein women’s bodies become sites for political and cultural regulation. Unlike interactions with the police or army discussed in the previous theme, paramilitary violence emerges in the testimonies of republican and loyalist women alike, as well as those unaffiliated with either community, emphasising the pervasive and indiscriminate nature of this violence.

### The Sexualisation and Victimisation of Female Combatants

In 6 of the 47 publications, women who were actively involved in the conflict as combatants reported their experiences of sexualisation and victimisation. Three descriptive themes emerged: (a) female combatants were sexualised; (b) female combatants were presumed to be promiscuous; and (c) female combatants became targets of sexual violence because they were combatants. See Table 7.

**Table 7.** The Sexualisation and Victimisation of Female Combatants.

Theme	References
The sexualisation and victimisation of female combatants	(Fairweather et al., 1984; Fitzpatrick, 2015; O’Keefe, 2013, 2017; Swaine, 2018; Van Ooijen et al., 2023)
Subthemes	References
Female combatants were sexualised	(Fairweather et al., 1984)
Female combatants were presumed to be promiscuous	(Fairweather et al., 1984; O’Keefe, 2013)
Female combatants became targets of sexual violence because they were combatants.	(Fitzpatrick, 2015; O’Keefe, 2013; Van Ooijen et al., 2023)

In general terms, and specifically in one of the included publications, female combatants said that they were sexualised because of their identity as a combatant. They were considered femmes fatales or ‘dangerous women,’ for example; British army cartoons also often portrayed them sexually, with captions that spoke of the “rapable terrorist” (Fairweather et al., 1984: 54).

Two of the included publications referred to the presumed promiscuity of female combatants. One interviewee reported that, when she joined the IRA, other IRA members assumed that she would be promiscuous and one male combatant assumed that she would “accompany him to his hotel room for the night.” (O’Keefe, 2013: 89).

While the woman did explain this incident away as “pub culture,” (O’Keefe, 2013: 89) she also highlighted that there were generally expectations or ideas around the sexual proclivities of female combatants involved in conflict as well as a sense of sexual entitlement from males.

In some cases, these attitudes led men to commit physical sexual violence against the women, because of or due to their active role in the conflict. Three of the included publications refer to such incidents. In one case, Anne, a former IRA quartermaster in Fitzpatrick (2015: 133), described an experience in a safehouse when her married commander “came on very strong” and she “wanted to say no but didn’t know how.” When she was later caught by the RUC, the officers then threatened to expose her sexual history with the married commander. “We are going to tell his wife.” (Van Ooijen, 2023: 1059).

The sexualisation and victimisation of female combatants during the Troubles reflect international patterns of CRSV, where perpetrators targeted women in militant roles because of their political affiliations and enforced gendered mechanisms of coercion and control. In this context, CRSV is used to reinforce male control and dominance in military hierarchies and conflict zones, functioning as a tool of punishment, humiliation, and subjugation (Seifert, 1994; Goldstein, 2001). During the Troubles, female combatants were perceived to be both political threats and sexually available, reinforcing narratives that conflated military participation with promiscuity. CRSV operated simultaneously as a tool of community control and a strategy for diminishing women’s autonomy in political movements. Recognising these gendered dynamics is essential for understanding how female combatants were not only participants in the conflict but also uniquely vulnerable to sexual violence, with lasting consequences for their ability to seek recognition, justice, and support. When women who were active in paramilitaries or were combatants experienced sexual violence, the perpetrator enjoyed an extra level of impunity. These women were unlikely to receive support from paramilitaries or the police, and many people in the wider communities also thought that militant women should expect to receive such treatment. Most of the testimony within this theme is contributed by republican combatants. Again, this is not to say that female combatants in loyalist paramilitaries were not present, nor that they did not experience sexualisation, but more likely that women in loyalist paramilitaries have been unable to speak out. McEvoy (2009) highlights that women’s roles in loyalist paramilitaries were often downplayed by male combatants, who also acted as gatekeepers in regard to researchers. As such, it is possible that this is another element where women’s and girls’ experiences of conflict may be missing from the narrative.

### Wider Impacts on Women, Girls, and the Community

In 44 of the included publications, women and girls spoke of the impact CRSV had on them and the wider community. Four descriptive themes emerged: (a) constant fear of becoming a victim of CRSV; (b) feelings during and after sexual assaults; (c) impunity and lack of support; and (d) normalisation of violence. See Table 8

**Table 8.** Wider Impacts on Women and the Community.

Themes	References
Wider impacts on women and the community	(Aretxaga, 1997, 2000, 2001; Belfast Women’s Collective, 1980; Belfrage, 1987; Brady et al., 2011; Calamati, 2002; Christian Response to Strip Search Group, 1997; Community for Justice, 1987; Corcoran, 2003; Corcoran, 2006; Fairweather et al., 1984; Faul and Murray, 1978; Fitzpatrick, 2015; Gallagher, 1992, 1995, 1997; Gilmartin and Scull, 2022; Green, 2019; Harris and Healy, 2001; London Strategic Policy Unit, 1988; Mairs, 2011; McAuley,1989; McCafferty, 1981; McVeigh, 1994; Moore, 2014; Mukungu, 2022; O’Keefe, 2013, 2017; Pickering and Third, 2003; Pickering, 2000, 2002, 2008; Shanahan, 1988; Shannon, 1997; Swaine, 2018; The London Armagh Group, 1984; Van Ooijen et al., 2023; Wahidin and Powell, 2017; Wahidin, 2016, 2018; Women Against Imperialism, 1980; Women in Struggle, 1992a, 1992b)
Subthemes	References
Constant fear of becoming a victim of CRSV	(Aretxaga, 2001; Belfast Women’s Collective, 1980; Brady et al., 2011; Calamati, 2002; Christian Response to Strip Search Group, 1997; Community for Justice, 1987; Fairweather et al., 1984; Faul & Murray, 1978; Fitzpatrick, 2015; Gallagher, 1992, 1995, 1997; Gilmartin & Scull, 2022; Green, 2019; Harris & Healy, 2001; Mairs, 2011; McAuley,1989; McCafferty, 1981; McVeigh, 1994; Mukungu, 2022; O’Keefe, 2013, 2017; Pickering, 2002; Shanahan, 1988; Shannon, 1997; Swaine, 2018; The London Armagh Group, 1984; Wahidin, 2016; Women in Struggle, 1992a, 1992b )
Feelings during and after sexual assaults	(Aretxaga, 1997, 2000, 2001; Belfast Women’s Collective, 1980; Brady et al., 2011; Calamati, 2002; Christian Response to Strip Search Group, 1997; Community for Justice, 1987; Corcoran, 2003; Corcoran, 2006; Fairweather et al., 1984; Faul and Murray, 1978; Fitzpatrick, 2015; Gallagher, 1992, 1995, 1997; Gilmartin and Scull, 2022; Green, 2019; Harris and Healy, 2001; London Strategic Policy Unit, 1988; Mairs, 2011; McAuley, 1989; McCafferty, 1981; McVeigh, 1994; Moore, 2014; Mukungu, 2022; O’Keefe, 2013, 2017; O’Keefe, 2017; Pickering, 2000, 2002, 2008; Pickering and Third, 2003; Shanahan, 1988; Shannon, 1997; The London Armagh Group, 1984; Van Ooijen et al., 2023; Wahidin, 2016, 2018; Wahidin and Powell, 2017; Women Against Imperialism, 1980; Women in Struggle, 1992a, 1992b)
Impunity and lack of support	(Harris and Healy, 2001; Mukungu, 2022; O’Keefe, 2013; Swaine, 2018; Van Ooijen et al., 2023)
Normalisation of violence	(Harris and Healy, 2001; McVeigh, 1994; Swaine, 2018; Van Ooijen et al., 2023; Waldhin, 2016)

*Note.* CRSV = conflict-related sexual violence.

Although CRSV was rarely discussed during the Troubles, its prevalence meant that many women and girls lived in constant fear of victimisation. This point was made in 31 of the included publications. In the words of Green (2019: 17):

You had this kind of enormous culture of violence which was created by the conflict which just sort of poured over into everywhere else. [It] poured over into the streets, into family life, into the home.

The encompassing nature of this violence caused women and girls in NI to feel extremely vulnerable and afraid, not just because of the control that paramilitary members exercised in their communities, but because of the power that state actors wielded. Fear of state actors was especially high because their use of force was perceived to be unquestioned and legitimate. Women and girls often found themselves confined in isolated settings with many men, which amplified their feelings of powerlessness and fear.

Just the sense of physical attack and I think the possibility of physical sexual attack makes women on their own very vulnerable, and very vulnerable too, in a situation when the police are never on their own; the police are always in a minimum of four. (Pickering, 2002: 151.)

The impact of CRSV during and after attacks was mentioned in 42 of the included publications. The women and girls described physical and psychological reactions:

[After the rape my] whole being sank in disgust, pain, embarrassment, grief, anger, frustration. (Van Ooijen et al., 2023: 1059.)

After a strip search, a former prisoner described her feelings in similar terms.

I feel so degraded but I won’t let them see that, it would only be worse for me. I try not to let my emotions break through, while inside is a mixture of revulsion and sickness at this act. (Community for Justice, 1987: 3.)

Five publications noted that the lack of support for victims and the impunity of perpetrators amplified the psychological impact of CRSV. The conflict permitted many men to exercise extraordinary levels of power and authority, which they exploited to minimise the consequences of their actions. For example, a service provider described the impunity of paramilitaries:

[T]here have been cover-ups of rapes. . . they do the pretence of saying “we are the protectors of the community; we will investigate this” and then take a young woman out of her house every night for a year and question her and try to get her to take back her story. (Swaine, 2018: 113–114.)

In five of the included publications, women and girls specifically mentioned that sexual violence was normalised during the conflict.

After a while abnormal situation becomes the norm of everyday life: so being stopped and searched was like the norm. There’s nothing normal about it. . . it was the norms of our Conflict. (Wahidin, 2016: 170.)

In NI [sexist harassment by the security forces] is probably an everyday incident experienced by a lot of people. No-one of any authority would take any notice. I don't feel that the security forces really care. (McVeigh, 1994: 156)

Harassment happens so often to people, they feel they won't be listened to - just like someone who was raped—they are afraid of others. (McVeigh, 1994: 171)

The discussion highlights the profound impact of CRSV on women, girls, and the wider community during the Troubles. Testimony within this theme came from women and girls across the political landscape, in both urban and rural areas, and from all age groups. The pervasive fear of CRSV, its psychological and physical effects, the impunity of perpetrators, and the normalisation of violence within communities highlight its deeper function as a tool of control and oppression. Nordås (2011) has demonstrated that both state and non-state actors frequently employ CRSV to reinforce dominance, silence protests, and shape post-conflict realities. In NI, these patterns of power, impunity, and suppression were evident in both state and non-state actions, as well as in the culture of silence that discouraged women and girls from reporting abuse or seeking justice. The long-term effects of CRSV are profound. They extend beyond individual trauma and shape entire communities. Nordås emphasises that CRSV can be both a deliberate wartime strategy and a broader mechanism of societal control, which forces populations to comply from fear and shame. Women’s and girls’ experiences reveal that the conflict resulted in direct violence but also shaped broader societal attitudes and allowed impunity and systemic harassment to persist. The normalisation of such violence meant that women and girls often felt unheard and unsupported, victims struggled to access compensation or justice, particularly in communities where perpetrators were celebrated as defenders or heroes. This has further exacerbated their trauma. (Tables 9, 10).

**Table 9.** Summary of Critical Findings.

Summary of Critical Findings.
• Sexual violence, the threat of violence and the power, control, and impunity brought about as a result of the conflict was a constant in women’s lives and was weaponised by both state and non-state actors. • This occurred in the women’s own homes, on the streets, at checkpoints, and in criminal justice interactions such as arrest, interrogation, and imprisonment. • While there is documentation of the harms faced by women during the Troubles, this is often not directly linked to concepts such as CRSV, meaning that it is left unaddressed

*Note.* CRSV = conflict-related sexual violence.

**Table 10.** Implications of the Review for Practice, Policy, and Research.

Implications of the review for practice, policy, and research
The review will inform policy and practice regarding mechanisms for dealing with the legacies of the past. Previous debate and policy approaches have not considered or addressed CRSV, nor all the experiences of women (Gallagher et al., 2012). This research therefore represents the viewpoints of an underrepresented population which can be used to inform and support submissions to oversight bodies including the NI Affairs Committee, Assembly Committees, the Victims Commission, and the CJS. Importantly, it would provide evidence of the need for a thematic strand in the Legacy Commission that would investigate sexual and gender-based violence, this currently absent in its remit/scope. In addition, the research will inform international discourse, research, policy approaches, and the (re)conceptualisation and framing of what constitutes CRSV.

*Note.* CRSV = conflict-related sexual violence.

## Conclusion

This research aimed to identify and interpret recurring themes of CRSV in NI during the Troubles, and to collect and analyse qualitative accounts of women and girls’ experiences. It sought to highlight the gaps within the field regarding victims’ experiences, addressing the continued absence of these narratives in both policy discussions and scholarly research. These gaps must be addressed to deepen understanding of the enduring harms that CRSV has caused to the women and girls directly affected, as well as to NI society more broadly, and to acknowledge and mitigate them.

For over 30 years, during the conflict, women and girl’s gender and sexuality were seen as a distinct “vulnerability . . . that’s the overbearing threat that you constantly face. . . It’s a threat that’s always there in a woman’s mind” (McVeigh, 1994: 127). Sex and gender were used and exploited in women and girls’ homes, in their communities, by other communities, by paramilitaries, and by state actors (such as the police and security forces). This became part of everyday life for women and girls in NI. Victims did not receive justice, and perpetrators were not prosecuted. As a result, for many people in NI, sexual violence, and particularly VAWG, was either invisible and silenced or normalised and expected. It contributed to the continuation of a patriarchal, violent and victim-blaming culture and to inherent structural inequalities such as political marginalisation, restrictive gender norms and economic instability.

‘Post-conflict’ NI continues to suffer from the legacy of “armed patriarchy.” As described by McLeod (2011), Cockburn (2013), Meintjes et al. (2001) and Gilmartin (2018), VAWG, and oppression and insecurity, often increase after a conflict ends. Adding to the statistics listed in the introduction, research by Anyadike-Danes et al. (2022) suggests that every second NI girl experiences sexual violence. Between 1991 and 1994, about one quarter of “ordinary, decent” murders were connected to domestic violence (McWilliams and Spence, 1996). NI post-conflict has the second-highest rate of femicide in Western Europe (FactcheckNI, 2019). Long after the rest of the United Kingdom, the NI executive finally introduced a strategic framework for ending VAWG in 2024 (NI Elects, 2024).

Policing of sexual violence in NI was and remains limited because resources were diverted to conflict-related issues, such as bombings and shootings (Swaine, 2015). Paramilitaries continue to act as ‘protectors,’ to exercise power and status in the community, and to enjoy impunity. The conflict enabled violent and abusive men to obtain power in paramilitary organisations (Doyle & McWilliams, 2018), and gain access to a wider variety of weapons, including guns (Montgomery & Bell, 1986). Even in ‘peacetime,’ membership of paramilitary organisations or state institutions (as the recent case of Sarah Everard highlighted in England) (Westmarland et al., 2021) confers power and influence in communities. Out of fear, many are reluctant to report instances of sexual violence by paramilitaries or police officers because they fear that the police ‘will do nothing’ or that they will be targeted in their community, or shamed, for speaking out. Swaine (2024) has emphasised that paramilitaries and paramilitary-affiliated men continue to exert coercive control in cases of intimate partner violence, demonstrating the impunity and power they still hold. Through this publication, the recurring themes of CRSV, its impact on women and girls, and its broader implications for NI society have been brought into focus. By identifying the gaps in research and policy, this systematic review has contributed to the growing discourse on CRSV and the structural inequalities that persist. Recognising these harms is a necessary step toward addressing them. This paper hopes to facilitate a more informed debate, both on how to mitigate the effects of CRSV and on designing policies that prioritise justice and protection for victims.

## Notes

1. “Taig” is an offensive or derogatory term for Catholics.

2. Tarring and feathering was a form of punishment in which women were often subjected to having their heads shaved, tied to lampposts, and covered with tar and feathers, serving both as physical punishment and public humiliation.

3. A “tout” is someone accused of being an informant or a snitch. In this context, it refers to passing information to, or collaborating with the RUC, British Army or other intelligence services.
